# Epidemiology, clinical features, and resource utilization associated with respiratory syncytial virus in the community and hospital

**DOI:** 10.1111/irv.12723

**Published:** 2020-02-20

**Authors:** Marie Smithgall, Philip Maykowski, Philip Zachariah, Matthew Oberhardt, Celibell Y. Vargas, Carrie Reed, Philip LaRussa, Lisa Saiman, Melissa S. Stockwell

**Affiliations:** ^1^ Department of Pathology and Cell Biology Columbia University Medical Center New York NY USA; ^2^ Department of Epidemiology Mailman School of Public Health New York NY USA; ^3^ Department of Pediatrics Columbia University New York NY USA; ^4^ NewYork‐Presbyterian Hospital New York NY USA; ^5^ Centers for Disease Control and Prevention Atlanta GA USA; ^6^ Department of Infection Prevention and Control New York‐Presbyterian Hospital New York NY USA; ^7^ Department of Population and Family Health Mailman School of Public Health Columbia University New York NY USA

**Keywords:** community surveillance, medically attended, respiratory syncytial virus

## Abstract

**Background:**

The epidemiology, clinical features, and resource utilization of respiratory syncytial virus (RSV) cases in the community and the hospital are not fully characterized.

**Methods:**

We identified individuals of all ages with laboratory‐confirmed RSV from two sources, a community cohort undergoing surveillance for acute respiratory infections (ARIs) and hospitalized patients from the same geographic area of New York City between 2013 and 15. The epidemiology, clinical features, and resource utilization (antibiotic/steroid/ribavirin usage, chest X‐rays, respiratory‐support (continuous positive airway pressure [CPAP], mechanical ventilation or extracorporeal membrane oxygenation [ECMO]), and indicators of disease severity (respiratory‐support, and/or ICU admission or death)) were compared among age groups using univariate and bivariate analyses.

**Results:**

In the community cohort (1777 people with 1805 ARIs), 66(3.7%) tested RSV‐positive (3.8% of <1‐year‐olds; 3.8% of adults ≥65); 40.9% were medically attended, and 23.1% reported antibiotic usage. Among 40,461 tests performed on hospital patients, 2.7% were RSV‐positive within ± 2 days of admission (37.3% <1 year old; 17.4% ≥65 years old). Among RSV‐positive hospitalized adults ≥65%, 92.7%, 89.6% and 78.1% received a chest X‐ray, antibiotics and/or steroids respectively, compared with 48.9%, 45.7%, and 48.7% of children <1. Severe illness occurred in 27.0% RSV‐positive hospitalized <1‐year‐olds and 19.8% ≥65‐year‐olds.

**Conclusions:**

Respiratory syncytial virus had a demonstrated impact in the community and hospital. Only 40% of RSV community cases were medically attended. In the hospitalized‐cohort, <1‐ and ≥ 65‐year‐olds accounted for the majority of patients and had similar rates of severe illness. In addition, resource utilization was high in older adults, making both young children and older adults important potential RSV vaccine targets.

## 1 INTRODUCTION

Respiratory syncytial virus (RSV) has been well‐characterized in children with an annual estimated 57 527 hospitalizations and 2.1 million outpatient visits for children <5 years in the US.^1^ However, recent studies have identified increased burden of RSV among adults ≥65 years old,^2^, ^3^, ^4^ especially those in contact with young children.^2^, ^5^ One model estimates that RSV accounts for approximately 10 000 deaths in ≥65‐year‐olds annually in the US^6^

While studies of RSV epidemiology have been conducted among medically attended cases,^1^, ^2^, ^7^, ^8^ a fuller understanding of RSV impact would be gained by examining infections and resource utilization in both the community and hospital setting in tandem specifically examining higher risk populations including adults ≥65 and young children. The objectives of this study were to use data from a community surveillance study of respiratory viral infections and from hospitalizations from the same geographic area to: (a) characterize RSV in a community cohort (including non‐medically attended cases) and hospitalized patients, (b) describe the clinical features and resource utilization of RSV, and (c) examine seasonal trends.

## 2 METHODS

### 2.1 Study design, study subjects, and study sites

A retrospective analysis was performed to identify individuals with laboratory‐confirmed RSV from January 2013 through December 2015. The community cohort consisted of prospectively enrolled participants in the Mobile Surveillance for acute respiratory infection (ARI)/influenza‐like illness (ILI) in the Community (MoSAIC) study (CDC 1U01IP000618) based in the Washington Height area of Northern Manhattan.^9^ Households in this study were primarily multigenerational, Latino and publicly insured. Briefly, households were queried twice‐weekly via text‐messaging for ARI symptoms. Mid‐turbinate nasal swabs were obtained at a home‐visit by research staff, the majority within 24‐48 hours of symptom onset, for those who meet symptomatic criteria‐defined as ≥2 of the following: fever/feverishness, cough, sore throat, runny nose/nasal congestion or body aches. Children <1 year old were also swabbed if they had rhinorrhea/congestion only.

The hospital population included patients admitted to the three hospitals in the MoSAIC community, all affiliated with NewYork‐Presbyterian Hospital. Patients were tested for respiratory pathogens using a respiratory viral panel (RVP) according to the medical judgment of their treating providers. Due to infection prevention and control virtually, all patients presenting with respiratory symptoms were tested. Hospitalized patients were identified based on RVP results and only included if their first positive RSV test was either within two calendar‐days prior to admission (eg, in the emergency department) or within the first two calendar‐days after admission to target primarily RSV‐associated hospitalizations.

For both community and hospital RSV detections, if a participant had >1 positive RSV test, subsequent episodes were included if detections occurred >4 weeks apart. The Columbia University Medical Center (CUMC) Institutional Review Board approved this study; the community cohort provided informed consent, a waiver of consent was granted for hospitalized patients.

### 2.2 Viral diagnostic testing

Mid‐turbinate nasal swabs from the community and nasopharyngeal swabs from hospitalized subjects were analyzed by multiplex RT‐PCR using the same FDA‐approved FilmArray Respiratory‐panel 1.7 (BioFire Diagnostics, Inc). The community samples were brought to one of the co‐investigator's laboratory (PSL) in universal transport media within 4 hours of collection for testing. The hospital samples were tested in the CUMC Clinical Microbiology Laboratory.

### 2.3 Clinical features and resource utilization in community and hospital subjects with RSV

For the community cohort, in addition to the ARI symptoms outlined above, these additional symptoms were captured: chills, fatigue, headache, wheezing, dyspnea, hoarseness, earache, conjunctivitis, rash, vomiting, diarrhea, and loss of appetite. On follow‐up calls conducted starting 10 days after illness report and continuing intermittently until symptom resolution, self‐reported visits (primary care, urgent‐care, Emergency Department and/or hospitalizations), antibiotic usage, and missed school/work days were collected to capture a summary of the entire illness. Index cases were defined as the first symptomatic household member.

The electronic medical records (EMR) of hospitalized patients positive for RSV were queried for select medications (antibiotics, steroids, albuterol, racemic epinephrine, and ribavirin) received from 2 days prior to 7 days after the positive RVP, chest X‐rays within ± 2 days of positive RVP, respiratory, blood and urine culture results within ± 2 days of positive RVP, type of respiratory‐support received including continuous positive airway pressure (CPAP), mechanical ventilation, extracorporeal membrane oxygenation (ECMO) and/ or ICU admission. Use of bilevel positive airway pressure (BiPAP) and oxygen supplementation was excluded as accurate data for their use could not be readily obtained in structured secondary sources. Blood and respiratory cultures were only considered positive if the identified species was a known pathogen (eg, *Pseudomonas aeruginosa*) and not a common contaminant (eg, *Staphylococcus epidermidis*).

### 2.4 Factors associated with increased severity of illness

In the community cohort, medically attended illness and missed school/work days were considered markers of more serious illness. Potential risk factors and co‐variates collected at enrollment included demographic characteristics (sex, race, ethnicity, language, age, and insurance), and chronic respiratory conditions.

Among hospitalized patients, severe illness was defined as respiratory‐support with CPAP, ventilation or ECMO, and/or ICU admission or death during the RSV hospitalization.^10^, ^11^ Patients’ International Classification of Diseases 9 and 10 (ICD) codes were queried to assess comorbid conditions and primary reason for hospitalization. Conditions were classified into chronic comorbid categories (CCC) tabulated by the study‐team based on consensus (Table S1) and included: cardiovascular, respiratory, endocrine, gastrointestinal, genetic/congenital neurologic including neuromuscular, renal, hematologic, transplant/immunosuppression, HIV, malignancy, metabolic, failure to thrive, obesity, prematurity (<37 weeks gestation in <2‐year‐olds), cystic fibrosis, and Down's syndrome.^4^, ^5^, ^10^, ^11^, ^12^, ^13^ If a specific condition was coded for <15 patients or did not fit one of the CCCs, it was disregarded.

The primary‐ICD diagnosis for each patient was categorized as respiratory or non‐respiratory by study‐investigators. There were 196 patients for whom there was no primary‐ICD code designated. For these patients, their additional ICD codes were examined to categorize the diagnosis as respiratory or non‐respiratory. For ambiguous diagnoses (eg, fever and dehydration), a chart‐review was conducted to assess the patient's primary symptoms for proper classification.

### 2.5 Data analysis

In the community cohort, bivariate analyses assessed the associations between demographic characteristics, viral co‐detections, and chronic conditions with more serious illness associated with RSV detection. The small number of illnesses detected precluded multivariate analysis.

Among hospitalized patients, bivariate analyses assessed the associations of demographic characteristics (eg, age, sex, race, ethnicity, age, primary language, and insurance), clinical factors (eg, primary respiratory diagnosis), CCCs (number and types) and presence of viral co‐detections with severe illness associated with RSV detection*.* Multivariable logistic regression analyses with forward‐selection were conducted to examine the associations between any variables positive in the bivariate analyses plus age (a priori inclusion*)*, and severe illness. Chi‐square test was conducted to examine the difference among clinical features and resource utilization (ie, chest X‐rays, antibiotic, and steroid usage) among age groups.

To assess seasonal trends, RSV activity overall and monthly was compared in the hospital and community groups. Epidemic curves and bivariate analysis were used to compare age groups (children <18 years vs adults ≥18 years) to month of illness onset or hospital admission. Analyses were conducted using SPSS v.23.

## 3 RESULTS

### 3.1 Study population

**Figure 1:**
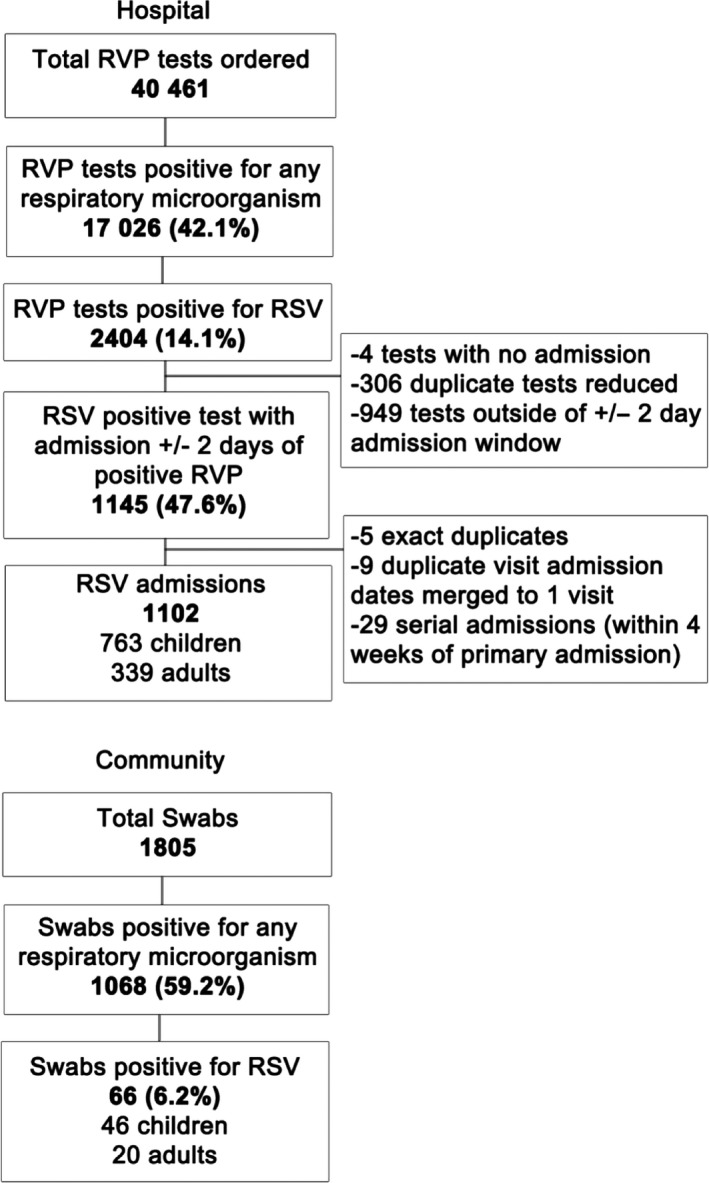
Method for inclusion in hospital and community study population based on respiratory viral panel (RVP) results

The community MoSAIC cohort consisted of 371 households (1777 participants, 53 children <1 year old, 716 children 1‐17 year old, 929 18‐64 year old, and 79 ≥65 year old), with an average of 4.8 members/household. Out of 1985 symptomatic ARI episodes reported during the study period, 1805 had a swab obtained for RVP testing and 66 (3.7%) tested positive for RSV [2 (3.8%) of <1‐year‐olds, 44 (5.7%) of 1‐17, 17 (1.8%) of 18‐64, and 3 (3.8%) of ≥65‐year‐olds] (Figure 1). Most RSV cases (78.8%) were within the RSV season (November‐February). The majority of RSV cases (68.1%) were female, Latino (100%) and publicly insured (78.8%) (Table 1). Few (18.2%) had chronic medical conditions, mostly (83.3%) asthma. Co‐detection of respiratory pathogens occurred for 21.2% of RSV‐positive participants, most commonly rhinovirus/enterovirus (64.3%). In the 9 households with multiple cases of RSV, school‐age children were the index case in all but one household.

**Table 1:** Demographic characteristics, chronic medical conditions, clinical features and resource utilization of the community cohort with RSV detected

Characteristic	Community participants with RSV detected (n = 66)
Age	
<1 y	2 (3.0)
1‐17 y	44 (66.7)
18‐64 y	17 (25.8)
≥65 y	3 (4.5)
Sex‐female	45 (68.1)
Race	
White	20 (30.3)
Other^a^	45 (68.1)
Unknown	1 (1.5)
Ethnicity	
Hispanic	66 (100)
Primary Language	
English	26 (39.4)
Spanish	21 (31.8)
NA	19 (28.8)
Insurance	
Private	9 (13.6)
Medicaid	52 (78.8)
Uninsured	3 (4.5)
Unknown	2 (3.0)
Self‐reported Health Status	
Excellent	16 (24.2)
Good	32 (48.5)
Fair	15 (22.7)
Poor	3 (4.5)
Hours outside home per week	
<10	25 (37.9)
11‐20	3 (4.5)
21‐40	19 (28.8)
>40	19 (28.8)
Chronic conditions	12 (18.2)
Asthma	10 (15.2)
Other Respiratory Disease	1 (1.5)
Diabetes Mellitus	4 (6.1)
Neurologic	1 (1.5)
Down's Syndrome	2 (3.0)
Non‐smoker	66 (100)
Co‐detection, Any (% with co‐detection)	14 (21.2)
Rhinovirus/ enterovirus	9 (64.3)
Coronavirus	5 (35.7)
*Chlamydophila pneumoniae*	2 (14.3)
Parainfluenza virus	1 (7.1)
Adenovirus	1 (7.1)
Antibiotics (completed course)	16 (24.2)
Medically attended	27 (40.9)
Patient/caregiver missed school/work	27 (40.9)
Total missed days	87
Average missed days per household	2.6 ± 2.0
Symptoms^b^	
Fever/Feverish	27 (40.9)
Cough	39 (59.1)
Sore Throat	19 (28.8)
Nasal Congestion	40 (60.6)
Body Aches	7 (10.6)
Dyspnea	1 (1.5)
Headache	6 (9.1)
Earache	3 (4.5)
Vomiting	2 (3.0)

a In the MoSAIC community, the majority of participants are Latino and some people identify this as both their race and ethnicity.

b None of the RSV‐positive participants reported chills, malaise/fatigue, wheezing, hoarseness, conjunctivitis, skin rash, diarrhea, or loss of appetite.

For patients hospitalized during the study period, 40,461 RVP tests were analyzed of which 17,026 (42.1%) were positive for any pathogen and 2404 (5.9%) for RSV. This study included the 1102 hospitalized patients (411 (37.3%) <1 year old, 352 (31.9%) 1‐17, 147 (13.3%) 18‐64, and 192 (17.4%) ≥65 year old) whose RVP was RSV‐positive within 2 days of admission (Figure 1). The majority (77.3%) of RSV cases were within the RSV season (November‐February). Just under half (43.4%) of RSV + hospitalized patients were Latino, and most were publicly insured (74.3%) (Table 2). Nearly a third of all hospitalized patients and half those ≥65 year old lived within the zip‐code catchment‐area of the community cohort study. Most patients (78.3%) had a primary respiratory diagnosis. A majority (60.4%) had had at least one CCC, with the highest percentage in adults ≥65 years old (96.9%) and lowest in children <1 year (28.2%)(*P* < .0001) (Table S1). Overall, 16.2% had a co‐detected respiratory virus, with the highest percent in young children <1 year old (19.0%) and the least (8.3%) among adults ≥65 years old (*P* < .0001), most commonly rhinovirus/enterovirus (56.2%) (Table 3).

**Table 2:** Demographic characteristics, chronic medical conditions, and clinical features of hospitalized patients with RSV detected

Characteristic	Hospitalized Patients with RSV Detected (n = 1102)
Age	
<1 y	411 (37.3)
1‐17 y	352 (31.9)
18‐64 y	147 (13.3)
65 y	192 (17.4)
Sex‐female	553 (50.2)
Race	
White	535 (48.5)
Black	151 (13.7)
Asian	15 (1.4)
Other	153 (13.9)
Unknown	248 (22.5)
Ethnicity	
Non‐Hispanic	307 (27.9)
Hispanic	478 (43.4)
Unknown	317 (28.8)
Primary Language	
English	634 (57.5)
Spanish	352 (31.9)
Other	102 (9.3)
Unknown	14 (1.3)
Insurance	
Commercial	225 (20.4)
Medicaid	619 (56.2)
Medicare	200 (18.1)
Uninsured	4 (0.4)
Unknown	54 (4.9)
MoSAIC Zip‐Code	345 (31.3)
Primary respiratory diagnosis	863 (78.3)
Number of chronic conditions	
0	437 (39.7)
1‐2	372 (33.8)
>3	293 (26.6)
Smoking	
Never	118 (10.7)
Current	35 (3.2)
Former	75 (6.8)
Unknown	874 (79.3)

**Table 3:** Resource utilization of hospitalized patients with RSV detected

Characteristic	Children < 1 (n = 411)	Children 1‐17 (n = 352)	Adults 18‐64 (n = 147)	Adults ≥ 65 (n = 192)	Overall (n = 1102)	*P*‐value
Co‐detection, Any (% with co‐detection)	78 (19.0)	69 (19.6)	15 (10.2)	16 (8.3)	178 (16.2)	<.0001
Rhinovirus/enterovirus	56 (71.8)	34 (49.3)	6 (40.0)	4 (25)	100 (56.2)	‐‐
Coronavirus	11 (14.1)	16 (23.2)	5 (33.3)	5 (31.3)	37 (20.8)	‐‐
Parainfluenza virus	5 (6.4)	7 (10.1)	0	1 (6.3)	13 (7.3)	‐‐
Adenovirus	6 (7.7)	10 (14.5)	0	0	16 (9.0)	‐‐
Influenza A (H3 and 2009H1)	1 (1.3)	4 (5.8)	4 (26.7)	4 (25)	13 (7.3)	‐‐
Influenza B	0	1 (1.4)	0	0	1 (0.6)	‐‐
Human metapneumovirus	3 (3.8)	2 (2.9)	0	3 (18.8)	8 (4.5)	‐‐
*Mycoplasma pneumoniae*	0	2 (2.9)	0	0	2 (1.1)	‐‐
Positive bacterial culture^b^	43 (10.5)	47 (13.4)	17 (11.6)	23 (12.0)	130 (11.8)	.578
Blood	10 (2.4)	10 (2.8)	8 (5.4)	4 (2.1)	32 (2.9)	‐‐
Urine	18 (4.3)	13 (3.7)	5 (3.4)	9 (4.7)	45 (4.1)	‐‐
Respiratory	15 (3.6)	24 (6.8)	4 (2.7)	10 (5.2)	53 (4.8)	‐‐
Chest X‐ray	201 (48.9)	250 (71.0)	124 (84.4)	178 (92.7)	753 (68.3)	<.0001
Medications						
Antibiotics	188 (45.7)	275 (78.1)	115 (78.2)	172 (89.6)	750 (68.1)	<.0001
Steroids	200 (48.7)	256 (72.7)	99 (67.3)	150 (78.1)	705 (64.0)	<.0001
Racemic epinephrine	176 (42.8)	66 (18.8)	0	2 (1.0)	244 (22.1)	‐‐
Ribavirin	0	1 (0.3)	12 (8.2)	6 (3.1)	19 (1.7)	‐‐
Oseltamivir	1 (0.2)	3 (0.8)	14 (9.5)	15 (7.8)	33 (3.0)	‐‐
Mean Length of Hospital Stay (d)	4.2 ± 5.8	4.9 ± 8.6	8.2 ± 12.9	6.6 ± 6.9	5.4 ± 8.3	‐‐
Respiratory readmission^a^	5 (1.2)	5 (1.4)	4 (2.7)	6 (3.1)	20 (1.8)	.103
Severe disease	111 (27.0)	86 (24.4)	22 (15.0)	38 (19.8)	257 (23.3)	.056
Continuous positive airway pressure	85 (20.7)	50 (14.2)	5 (3.4)	19 (9.9)	159 (14.4)	.001
Ventilator	14 (3.4)	10 (2.8)	7 (4.8)	15 (7.8)	46 (4.2)	.018
Extracorporeal membrane oxygenation	1 (0.2)	2 (0.6)	1 (0.7)	0	4 (0.4)	‐‐
ICU admission (≥1)	63 (15.3)	64 (18.2)	21 (14.3)	25 (13.0)	173 (15.7)	.455
Mean(SD) ICU‐hours	148.0 ± 219	179.3 ± 232	257.0 ± 267	181.6 ± 153	177.8 ± 223	‐‐
Case fatality ratio^a^	1 (0.2)	2 (0.6)	3 (2.2)	11 (5.9)	17 (1.6)	‐‐

Note

Contaminants: *Ochrobactrum anthropi*, *Staphylococcus capitis*, *Staphylococcus epidermidis*, *Staphylococcus hominis*, *Streptococcus agalactiae*, *Streptococcus mitis*, *Streptococcus salivarius*, *Streptococcus vestibularis*, *Streptococcus viridian.*

a Within 4 wk of discharge.

b Pathogenic organisms (bolded organisms isolated from blood cultures): *Acinetobacter baumannii*, *Enterobacter cloacae*, *Enterococcus faecalis*, *Escherichia coli*, *Klebsiella oxytoca*, *Klebsiella pneumoniae*, *Moraxella catarrhalis*, *Proteus mirabilis*, *Pseudomonas aeruginosa*, *Serratia marcescens*, Methicillin‐resistant *Staphylococcus aureus*, Methicillin‐susceptible *Staphylococcus aureus*, *Stenotrophomonas maltophilia*, *Streptococcus pneumonia.*

### 3.2 Associated clinical features and resource utilization of RSV cases

In the community cohort, 40.9% of participants had medically attended visits, mostly to a primary care doctor's office (96.4%). One participant reported an emergency room visit and there were no reported hospitalizations. Nearly a quarter of all participants with RSV (24.2%) reported taking antibiotics with 87.5% reported having been prescribed the antibiotics by a doctor. The remainder reported getting antibiotics from a local store without a prescription or not remembering from where they obtained the antibiotics. Only half of those taking antibiotics reported being diagnosed with a potential bacterial infection such as otitis media. A total of 27 participants (40.9%) reported that either the sick participant and/or a caregiver had missed school/work with an aggregated total of 87 missed days (average 2.6 days/case). The most commonly reported symptoms across all ages included nasal congestion (60.6%), cough (59.1%), and fever/feverishness (40.9%) (Table 1).

Among hospitalized patients, children <1 were more likely to be treated with CPAP (20.7%) vs patients ≥65 years (9.9%) (*P* = .001). However, ≥65‐year‐olds were more likely to receive respiratory‐support with mechanical ventilation vs children <1 (7.8% vs 3.4%) (*P* = .018). Nearly 16% of all patients had at least one admission to the ICU. Overall, 20 patients (1.8%) had a respiratory readmission within 4 weeks of discharge; (Table 3).

Among those ≥65 years old, 92.7% had a chest X‐ray, and 89.6% and 78.1% received antibiotics and/or steroids, respectively. Among children <1 year, 48.9% had a chest X‐ray, and 45.7% and 48.7% received antibiotics and/or steroids, respectively (Table 3). Adults 18‐64 had the highest percentage of ribavirin administration (8.2%) compared with 3.1% in adults ≥65 and 0% in children <1. Patterns were similar when the sample was restricted to those from the catchment‐area.

### 3.3 Factors associated with increased severity of illness

In the community cohort, children and adults ≥65 were more likely to have a medically attended illness including 100% of children <1 year (n = 2), 52.3% of children 1‐17 years (n = 23) and 33.3% ≥65‐year‐olds (n = 1) compared with 5.9% of adults 18‐64 (n = 1) (*P* < .0001). Children or their caregivers were most likely miss school or work (100% of children <1 (n = 2), 54.5% of children 1‐17 (n = 24) vs those 18‐64 (11.8%), *P* = .025). One of the adults ≥65 (33.3%) missed two days of work. Other demographic factors and presence of CCCs were not associated with medically attended illness or missed school/work.

Among hospitalized patients, 111 (27.0%) <1‐year‐olds, 86 (24.4%) 1‐ to 17‐year‐olds, 22 (15.0%) 18‐ to 64‐year‐olds, and 38 (19.8%) ≥65‐year‐olds had severe illness. After adjusting for sex, race and age, having a genetic/congenital disease (aOR 4.1, CI 1.01‐16.5) as well as having ≥1 CCC (aOR: 2.9, 1.8‐4.8) increased the odds of severe illness in <1‐year‐olds. After adjusting for sex, race and age, neurologic disease (aOR: 9.4, 2.8‐31.4), respiratory disease (aOR: 6.1, 2.6‐14.4), and congestive heart failure (aOR: 3.0, 1.3‐7.0) increased the odds of severe illness in those ≥65 years old (Table 4).

**Table 4:** Factors associated with increased severity of illness in hospitalized patients

	Children < 1 (n = 411)				Adults ≥ 65 (n = 192)			
	Severe Disease		OR 95% CI	aOR 95% CI	Severe Disease		OR 95% CI	aOR 95% CI
	Yes	No			Yes	No		
Congestive Congestive Heart Failure	‐‐	‐‐	‐‐	‐‐				
Yes					23 (31.1%)	51 (68.9%)	3.1	3.0
No					15 (12.7%)	103 (87.3%)	1.5‐6.4	1.3‐7.0
Respiratory disease				‐‐				
Yes	15 (36.6%)	26 (63.4%)	1.6		19 (47.5%)	21 (52.5%)	6.3	6.1
No	96 (25.9%)	274 (74.1%)	0.8‐3.2		19 (12.5%)	133 (87.5%)	2.9‐13.9	2.6‐14.4
Neurologic disease				‐‐				
Yes	6 (66.7%)	3 (33.3%)	5.7		9 (52.9%)	8 (47.1%)	5.7	9.4
No	105 (26.1%)	297 (73.9%)	1.4‐23.0		29 (16.6%)	146 (83.4%)	2.0‐15.9	2.8‐31.4
Genetic/congenital Disease					‐‐	‐‐	‐‐	‐‐
Yes	8 (72.7%)	3 (27.3%)	7.7	4.1				
No	103 (25.8%)	297 (74.3%)	2.0‐29.5	1.01‐16.5				
≥1 CCC								
Yes	50 (43.1%)	66 (56.9%)	2.9	2.9	‐‐	‐‐	‐‐	‐‐
No	61 (20.7%)	234 (79.3%)	1.8‐4.6	1.8‐4.8				

Abbreviations: aOR, adjusted odds ratio; CCC, Chronic comorbid condition; CI, confidence interval.

### 3.4 Respiratory syncytial virus epidemiology

**Figure 2:**
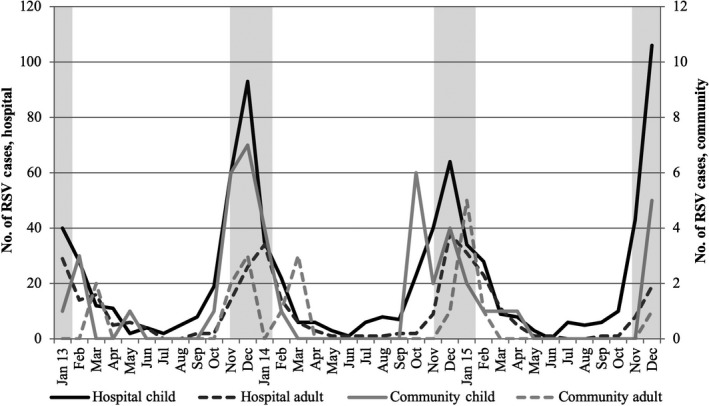
Respiratory syncytial virus (RSV) cases in children and adults in the community and hospital. Shaded bars indicate CDC predicted New York RSV seasonality window (mid‐November to February)

Both hospital and community RSV‐detections began in the early fall, peaked during the end of November/early December and tapered off by spring, without lag time between RSV in the community and hospital (Figure 2). Hospital detections of RSV occurred year‐round among children; however, none was detected among hospitalized adults nor in the community cohort during the summer months. The subsequent resurgence of detections in the community corresponded to the rise in hospital detections in the early fall. This pattern was similar when restricting to those from the catchment‐area. There was a significant difference between timing of RSV detected in adults (≥18 years) and children (<18 years) at both the hospital and community levels (*P* = .001) (Figure 2). While RSV was detected consistently beginning in September in children, RSV in adults was detected one month later on average.

## 4 DISCUSSION

With complementary datasets, this study examined RSV infection at the community and hospital levels highlighting the greater impact of more severe RSV in the oldest and youngest age groups as well as allowing for direct comparison of clinical features, resource utilization, and epidemiology of these higher‐risk age groups. In the community, though there were no hospitalizations, RSV demonstrated healthcare and economic burden as illustrated by the proportion of cases with medical attention (40.9%) and antibiotic use (23.1%), which is similar to the level of medical attention for influenza in this community cohort^9^, ^14^ and more than that of other viral respiratory pathogens.^9^, ^15^ In addition, RSV illnesses led to a high proportion of ill participants or their caregivers (40.9%) missing school or work, which can have a substantial financial impact, especially on people living in a low‐income community. Among hospitalized patients most infections were among children (<1 year) and older adults (≥65 years).^1^, ^2^, ^5^ Older hospitalized adults demonstrated high morbidity and had the highest resource utilization, despite having a similar risk of severe disease as young children. Therefore, this study highlights the substantial impact RSV can have on older adults.

In addition, we found factors that increased the risk of severe illness among the two highest risk populations. For children <1 year old, having a genetic/congenital condition as well as having ≥1 chronic condition increased the risk of severe illness. Among adults ≥65, chronic respiratory disease and congestive heart failure were also associated with severe illness. These findings are consistent with previous studies.^1^, ^2^, ^4^, ^16^ New in this study was the link between neurologic disease and severe RSV illness in adults ≥65; previously neurologic disease had been implicated in increased risk of pneumonia and acute respiratory infections overall but not RSV.^17^

This study also examined RSV seasonal trends from January 2013 through December 2015. Data collected by the National Respiratory and Enteric Virus Surveillance System (NREVSS) and analyzed by the CDC place New York's RSV seasonality window as mid‐November to February.^18^ Both our community and hospital data exhibited peak infections during this window for three consecutive years. However, recent NREVSS data suggest that surveillance using PCR assays as opposed to antigen based testing demonstrates an earlier onset of RSV detection both nationally as well as in New York.^19^ Similarly, we saw the initial rise of RSV infections beginning well before the projected start to the RSV season.^20^ For children, detection began to increase in September and for adults in October. While studies suggest that adults often acquire RSV from children^21^ and in our study most index cases in the community cohort were among school‐age children further emphasizing their potential role in spreading RSV, other congregate settings such as nursing homes or public gatherings could contribute to later RSV detection in adults.^5^ The AAP recommends administering Palivizumab, a monoclonal antibody, to high risk infants and young children for 5 months during the RSV season, beginning in November.^22^ Based on our data, severe disease cases peaked in mid‐November and early December, suggesting that increased surveillance of RSV cases in New York may be warranted to help with Palivzumab timing. In addition, given the high cost and burden of administering Palivzumab monthly, a barrier to widespread use, work toward an RSV vaccine remains important. The disproportionate burden of severe RSV illness among both young children and older adults highlight two potential target populations for RSV vaccination.

There are limitations in this study. The community cohort was primarily Latino and lived in a low‐income, urban neighborhood; thus, the community findings may not be generalizable to a broader population. Also, the clinical features and resource utilization of the community cohort were captured solely by interview and could not be verified by medical‐record documentation. While the hospital and community cohort are in the same geographic area, no one from the community cohort was hospitalized during the study period, potentially due to the relatively smaller sample size. In addition, findings from the hospital group likely reflect referral center bias; however, patterns were similar when assessing just those from the catchment‐area. Also, due to EMR limitations, we were unable to abstract accurate data on all types of respiratory‐support, calculate percent positive by age or analyze the results of chest X‐rays in the hospitalized group. In addition, by narrowing our selectivity to RSV‐positive RVP within ± 2 days of admission and collecting data on resource utilization with ± 2 days of positive RVP, we may have underestimated the true burden of RSV among hospitalized patients. Finally, our small sample size for community cases limited our ability to analyze adults ≥65 years old.

In conclusion, this study suggests that hospitalization with RSV disproportionately affects children <1 year old as well as adults ≥65 as judged by comparing the age distribution of cases with that in a community cohort. In addition, among hospitalized patients, severe illness associated with RSV mostly affected adults ≥65 and children <1 year old; however, resource utilization in the hospital was highest for older adults. Thus making, both older adults and young children important targets for RSV vaccines. Furthermore, trends suggest that in New York City, RSV cases in both the inpatient and outpatient setting are beginning earlier than previously anticipated.

## CONFLICT OF INTERESTS

The authors have no conflict of interest to declare. L. Saiman and P. LaRussa receive funding from Merck for a project related to RSV and MS. Stockwell is an unfunded investigator. The work detailed in this manuscript pre‐dates that funding.
