# Supplementary material for: Epidemiology, clinical features, and resource utilization associated with respiratory syncytial virus in the community and hospital
Source: Influenza Other Respir Viruses. 2020 Feb 20;14(3):247–56. doi: 10.1111/irv.12723 (PMC7182604; doi:10.1111/irv.12723)
Supplement: Supplementary file 1 [file IRV-14-247-s001.docx]

Supplemental Table 1: Chronic Conditions of Hospitalized Patients with RSV detected

| Chronic conditions | Children <1 (n=411) | Children 1-17  (n=352) | Adults 18-64 (n=147) | Adults ≥65  (n=192) | Overall  (n=1102) |
| --- | --- | --- | --- | --- | --- |
| Cardiovascular | 13 (3.2) | 27 (7.7) | 35 (23.8) | 82 (42.7) | 157 (14.2) |
| Congenital Heart Disease | 33 (8.0) | 33 (9.4) | 3 (2.0) | 1 (0.5) | 70 (6.4) |
| Congestive Heart Failure | 1 (0.2) | 3 (0.8) | 19 (12.9) | 74 (38.5) | 97 (8.8) |
| Hypertension | 4 (1.0) | 7 (2.0) | 40 (27.2) | 92 (47.9) | 143 (13.0) |
| Respiratory | 41 (10.0) | 52 (14.8) | 23 (15.6) | 40 (20.8) | 156 (14.2) |
| Chronic obstructive pulmonary disease (COPD) | 2 (0.5) | 3 (0.9) | 10 (6.8) | 37 (19.3) | 52 (4.7) |
| Asthma | 7 (1.7) | 124 (35.2) | 48 (32.7) | 37 (19.3) | 216 (19.6) |
| Neurological | 9 (2.2) | 34 (9.6) | 4 (2.7) | 17 (8.9) | 64 (5.8) |
| Dementia/Alzheimer’s | 0 | 0 | 0 | 32 (16.7) | 32 (2.9) |
| History of Stroke | 0 | 4 (1.1) | 5 (3.4) | 33 (17.2) | 42 (3.8) |
| Hematologic | 3 (0.7) | 3 (0.9) | 6 (4.1) | 7 (3.6) | 19 (1.7) |
| Anemia | 7 (1.7) | 13 (3.7) | 29 (19.7) | 42 (21.9) | 91 (8.2) |
| Sickle Cell Disease | 2 (0.5) | 8 (2.3) | 2 (1.4) | 0 | 12 (1.1) |
| Gastrointestinal | 1 (0.2) | 11 (3.1) | 4 (2.7) | 5 (2.6) | 21 (1.9) |
| Gastroesophageal Reflux Disease (GERD) | 15 (3.6) | 12 (3.4) | 22 (15.0) | 31 (16.1) | 80 (7.3) |
| Endocrine | 2 (0.5) | 5 (1.4) | 7 (4.8) | 24 (12.5) | 38 (3.4) |
| Diabetes | 0 | 1 (0.3) | 48 (32.7) | 85 (44.3) | 134 (12.2) |
| Renal | 2 (0.5) | 4 (1.1) | 32 (21.8) | 62 (32.3) | 100 (9.1) |
| Transplant/immunosuppression | 1 (0.2) | 14 (4.0) | 15 (10.2) | 9 (4.7) | 39 (3.5) |
| HIV | 0 | 0 | 5 (3.4) | 1 (0.5) | 6 (0.5) |
| Malignancy | 0 | 4 (1.1) | 24 (16.3) | 59 (30.7) | 87 (7.9) |
| Metabolic | 1 (0.2) | 2 (0.6) | 2 (1.4) | 5 (2.6) | 10 (0.9) |
| Failure to thrive | 12 (2.9) | 16 (4.5) | 0 | 0 | 28 (2.5) |
| Obesity | 1 (0.2) | 2 (0.6) | 20 13.6) | 17 (8.8) | 40 (3.6) |
| Prematurity | 8 (1.9) | 2 (0.6) | 0 | 0 | 10 (0.9) |
| Cystic Fibrosis | 1 (0.2) | 3 (0.9) | 4 (2.7) | 0 | 8 (0.7) |
| Down’s Syndrome | 4 (1.0) | 10 (2.8) | 1 (0.7) | 0 | 15 (1.4) |
| Genetic/Congenital | 11 (2.7) | 13 (3.7) | 2 (1.4) | 3 (1.6) | 29 (2.6) |
